# Incidence of Radix Entomolaris in Mandibular First Molars in Palestinian Population: A Clinical Investigation

**DOI:** 10.1155/2014/405601

**Published:** 2014-11-24

**Authors:** Raed Mukhaimer, Zafer Azizi

**Affiliations:** ^1^Department of Conservative Dentistry, Arab American University, Jenin, Palestine; ^2^Department of Pediatric Dentistry, Arab American University, Jenin, Palestine

## Abstract

*Purpose*. The aim of this investigation was to evaluate clinically the percentage of permanent mandibular first molar teeth with three roots amongst Palestinian population. *Patients and Methods*. Three hundred twenty-two mandibular first molars from 185 females and 137 males scheduled for root canal treatment at the Dental Center of the Arab American University were examined over a 2-year period. The incidence of a third root revealed by periapical radiographs and the comparison of the occurrence between males and females and between the right and left sides of the mandible were recorded. *Statistical Analysis*. It was performed using the chi-square test with a significant level set at *P* < 0.05%. *Results*. Of the 322 treated mandibular first molars, twelve teeth were found to have a third root with an overall incidence being 3.73%. More teeth with a third root were treated on the right side of the mandible compared to the left side. *Conclusion*. The incidence of a third root in Palestinian population was within the range of previous reports from the Middle East but considerably lower than the percentage from the Far East.

## 1. Introduction

Successful outcomes of endodontic treatment depend on the identification of all roots and root canals which in turn guarantees complete extirpation of pulp tissue, proper chemomechanical cleaning and shaping and three-dimensional obturation of the root canal system with an inert filling material [1]. Failure of at least one of these stages entails high risk of unsuccessful root canal treatment of the tooth with a subsequent development or persistence of a periapical lesion [2].

Normally, mandibular first molars have one mesial root and one distally. The mesial root has two canals (mesiobuccal and mesiolingual), ending mainly in two distinct apical foramina. Sometimes, these merge together to end in one foramen. The distal root typically has one root canal, although if the orifice is particularly narrow and round, a second distal canal may be present. Anatomical variations in the number of roots as well as canal configuration in mandibular molars are not uncommon [3, 4].

One of the major anatomical variations is the presence of an additional third root, also called the radix entomolaris (RE) which is located distolingually in mandibular molars. In very rare cases, when this additional root is located mesiobuccally, it is called radix paramolaris [5, 6].

Anatomical studies have reported an association between the presence of a separate RE in the first mandibular molar and certain ethnic groups. In populations with Mongoloid traits, such as Chinese, Eskimos, and American-Indians, it occurs with a frequency of 5 to more than 30% [7, 8]. In African population, a maximum frequency of 3% was found [9, 10], whereas in Europeans the incidence was even less. Schäfer et al. [11] using full-mouth periapical radiographs, investigated the incidence of radix entomolaris in German population. A total of 1,024 mandibular first molars were evaluated. Left molars comprised 500 teeth and right molars 524 teeth. Seven patients were found to have a three-rooted mandibular first molar with an overall incidence of 1.35%.

In Indian population, Garg et al. examined 1054 periapical radiographs and reported 5.97% of occurrence of RE in mandibular first molars [12]. The same method was used by Karale et al. [13] who reported a higher incidence (6.67%) of RE.

Knowledge of occurrence, location, and incidence of any tooth anatomical variation is important as it has a significant role in clinical dentistry. Many epidemiological studies have highlighted the importance of watching RE while performing root canal treatment on mandibular first molars. However, data is rare in relevance to Palestinian population. Dental schools have been very recently established in Palestine and there is no single research dealing with teeth anatomy and the incidence of anatomical variation in our country. With a huge number of dentists and very few specialists in endodontics, this research has been conducted so as to provide information about various anatomical variations that could be encountered during endodontic therapy. The purpose of this study was to evaluate the percentage of permanent mandibular first molar teeth with three roots in a Palestinian population using conventional digital X-rays in two different angles.

## 2. Patients and Methods 

Three hundred and twenty two mandibular first molars from 185 females and 137 males of different ages scheduled for root canal treatment at the Dental Center of the Arab American University were included in this clinical investigation. The study sample represents all patients who needed primary root canal treatment or referred for retreatment over a 2-year period. The age of the 322 participants ranged from 11 to 62 (mean = 37) years. All teeth treated had fully formed roots. The selected patients were explained about the proposed treatment and its criteria for evaluation. This study was approved by the “Ethics Committee” of the School of Dentistry Research Centre. Written consents were obtained from all the study participants. The criteria used to indicate the presence of RE were clear distinction of an extra root, indicated by the crossing of translucent lines defining the pulp space and periodontal ligaments, originating in the upper half of the distal root.

One endodontist and one paediatric dentist (both instructors of undergraduate dental students) served as examiners in this study. Disagreement in the interpretation of the radiographs was discussed between the two investigators until a consensus was reached. At least two preoperative radiographs were taken for each tooth undergoing root canal treatment using a digital X-ray sensor (Dr. Suni, San Jose, California, USA). One radiograph was taken from orthoradial position and the other taken either 30° mesially or distally. When the radiographs revealed a case of RE, another radiograph for the opposing side was taken.

After obtaining adequate anaesthesia, the tooth was isolated with rubber dam and root canal treatment was initiated. Conventional access cavity preparation was completed. The pulp chamber was irrigated with 3% sodium hypochlorite and carefully examined with an endodontic probe (DG-16, Dentsply, Gloucester, UK). All canals were scouted using K-file number 10 (Dentsply, Maillefer, Ballaigues, Switzerland). Working length was estimated using an apex locator (NovApex, Forum Technologies, Rishon Le-Zion, Israel) and confirmed with a working length radiograph with K-files introduced into the canals.

**Figure 1:**
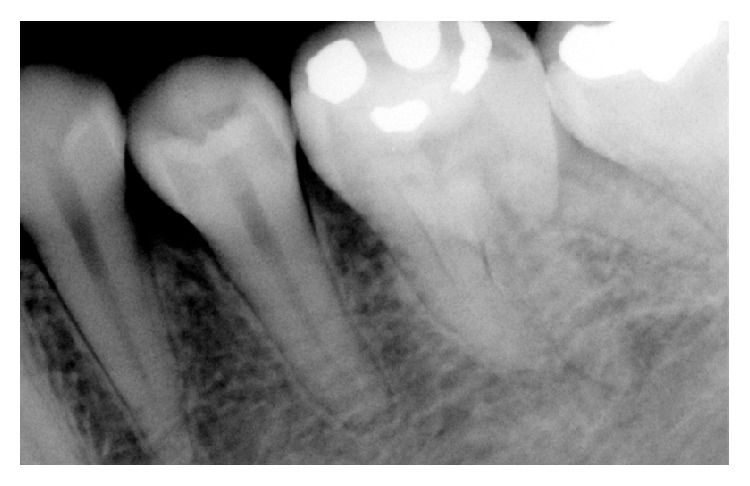
Preoperative radiograph showing three separate roots of mandibular first molar.

**Figure 2:**
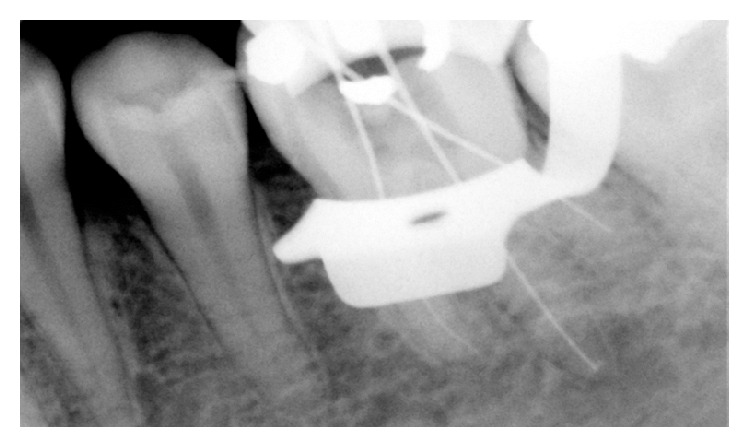
Radiograph of working length with three K-files in MB, DB, and DL root canals.

**Figure 3:**
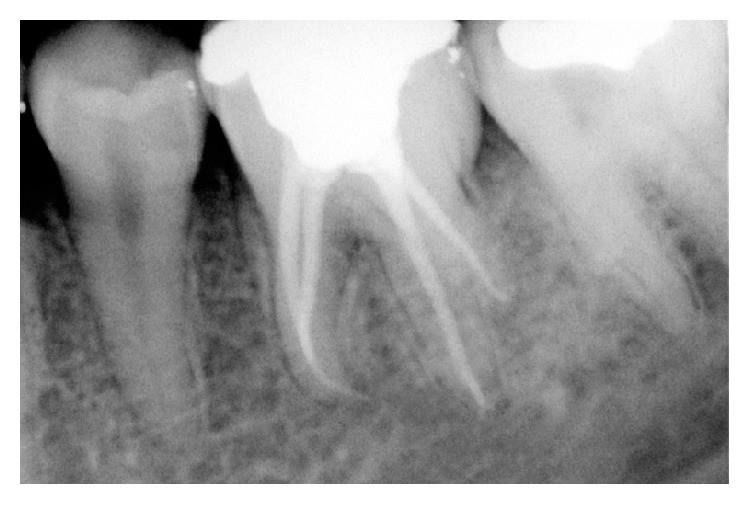
Postoperative radiograph of obturation.

After complete cleaning and shaping, all canals were obturated using lateral condensation technique. AH plus (Dentsply DeTrey, Konstanz, Germany) was used as a sealer. A postoperative radiograph was taken to assess the technical quality of root canal filling. When satisfactory, a permanent filling was placed. Figures 1, 2, and 3 show an example of a mandibular first molar with three roots.

The incidence of RE and comparison of the occurrence between males and females and between the right and left sides of the mandible were recorded.

Comparison of the incidence and the correlations between males and females and left- and right-side occurrences were analyzed by using the Pearson chi-square test with SPSS (15.0; SPSS Inc., Chicago, IL, USA). *P* < 0.05 was considered statistically significant.

## 3. Results 

Three hundred and twenty two patients comprising 185 females and 137 males formed the study sample. Twelve teeth were found to have RE with an overall prevalence being 3.73%. There was no significant difference in the incidence of three-rooted mandibular first molars between females (7/185) and males (5/137) (Table 1). However, there was a significant difference between the right side (8/12) and the left side (4/12) (*P* < 0.05). The bilateral incidence of symmetrical distribution was 33.3% (4 of 12).

## 4. Discussion 

Knowledge of both normal and abnormal anatomy of teeth dictates the parameters for execution of root canal therapy and can directly affect the probability of success. Therefore, practitioners should be familiar with the existence as well as the prevalence of teeth abnormalities [14]. Mandibular first molars seem to be the most frequent teeth in need of root canal treatment as they are the first permanent teeth to erupt. Nonetheless, anatomical variations of the root canal system in molars are not appreciated by a great number of general practitioners [15].

The presence of a third root (RE) may complicate the endodontic treatment and lead to failure as a result of canal missing. While conducting root canal treatment in mandibular first molars, clinicians should be aware of this morphological abnormality. De Moor et al. [16] have classified RE evaluated from extracted teeth into three types. Type I refers to straight root or canal. Type II refers to an initially curved entrance which continues as a straight root/root canal. Type III refers to an initial curve in the coronal third of the root canal and a second curve beginning in the middle and continuing to the apical third.

Accurate clinical and radiographic examination should be performed before initiating a root canal treatment. The infrequent occurrence of RE requires that the clinician be cautious in diagnosis and management of the lower molar teeth. Visual inspection of the tooth crown can facilitate identification of an additional root. Although it is not necessary, an additional root is often associated with an increased number of cusps and an increased number of root canals with a more prominent occlusodistal or distolingual lobe [17].

Careful radiographic diagnosis plays a pivotal role in endodontic treatment. Radiographs taken at different angulations reveal the basic information regarding the anatomy of a tooth and can thus help to detect any aberrant anatomy such as extra canals and/or roots [16]. When the outline of the distal root or the root canal seems unclear on the preoperative radiograph, the presence of a “hidden” third root should be suspected. Studies have shown that a second radiograph should be taken from a more mesial or distal angle (30 degrees) which could probably reveal the presence of RE [18].

Periapical radiographs were used in this study because they are routinely used in the Dental School of the Arab American University throughout endodontic steps. This technique is noninvasive and inexpensive and allows for interstudy comparisons relating to gender and bilateral occurrence difference for three-rooted mandibular first molars. On the other hand, this method has some disadvantages. As radix entomolaris is mostly situated in the same buccolingual plane as the distobuccal root, a superimposition of both roots can appear on the radiograph resulting in an inaccurate diagnosis [19].

Digital radiography was used in this study. The digital system offers many advantages over the conventional radiography such as ease and speed of use, reduction in time between exposure and image interpretation, less radiation dosage to the patient, elimination of chemical waste hazard, and the ability to digitally manipulate the captured image [20]. Unfortunately, a more advanced technology represented by the Cone Beam Computed Tomography (CBCT) is not available. Cone beam computed tomography scans were recently shown to be a valuable tool in several stages of endodontic treatment as they provide an immediate and accurate three-dimensional radiographic image. Preoperatively, these images give information about the internal and external tooth anatomy which include number and location of roots and canals, root and canal curvatures, size of the pulp chamber, and the degree of calcification [21]. CBCT images allow a complete elimination of the superimposition of structural images outside the area of interest and provide a high-contrast resolution and data from a single computed tomography imaging process. They also provide three dimensional images in the axial, coronal, or sagittal planes [22]. In cases of RE, CBCT shows the exact position of distolingual root and hence it helps in tracking the curvature and prevents iatrogenic event that might occur in relation to canal curvature like instrument separation, perforation, ledge formation, and so forth.

When the occurrence of RE is confirmed or suspected on the radiograph, the access cavity preparation should be modified from the classic triangular access to a more rectangular or trapezoidal outline. The orifice of RE is mainly located disto- to mesiolingually from the main distal canal. If the entrance of RE canal is not clearly visible after removal of the pulp chamber roof, a more thorough inspection of the pulp chamber floor and wall, especially in the distolingual region, is necessary. A sharp endodontic explorer (DG-16) can be useful in this respect [19].

The introduction of dental operating microscope (DOM) has changed the face of endodontics. Although the operating microscope is not used in our clinics, its use is recommended in routine endodontic practice as it offers an excellent illumination and magnification of the operating field and provides a tremendous advantage in locating and treating “extra” canals. Coelho de Carvalho and Zuolo [23] reported that the DOM had enabled them to locate 8% more canals in mandibular molars.

Al-Nazhan has examined 251 mandibular first molars of Saudi patients (clinically and radiographically). He reported an incidence of 6% of RE amongst Saudi population [24]. In their clinical investigation conducted on Chinese population, Yu et al. screened 378 cases of mandibular first molars with root canal therapy and reported an incidence of 27% of teeth with RE [25].

In our study, the overall incidence of patients with three-rooted mandibular first molars was 3.73%. This finding was in a range of previous reports on Middle Easterners [24, 26]. It was also close to reports from India [12, 13]. However, it was low when compared with data reported for Asian races: 24.5% in Koreans [27], 32% in Chinese [28], and 25.6% in Taiwanese [29]. In the present study, no significant difference in the incidence of RE was found between males and females. The same result was reported by other studies [11, 12, 28, 30].

When considering the right and left sides of the mandible, three-rooted mandibular first molars occurred more frequently on the right side than on the left side. These findings were in accordance with some previous studies [12, 28] and different from some others [11, 31] which reported that RE can occur more on the left side. Çolak et al. [30] and Yang et al. [32] found no significant difference between both sides.

Studies done by De Moor et al. [16] and Loh [33] report a bilateral occurrence of three rooted mandibular permanent first molars from 50 to 67%; however, in our study the bilateral occurrence was only 33.3%. This observation was similar to the findings of Çolak et al. [30]. This percentage was higher than the study on German population [11] and much lower than the studies [27–29] involving Asian subjects (Koreans, Chinese, and Taiwanese, resp.).

## 5. Conclusion

General practitioners as well as specialists in endodontics should always think about a possible third root (RE) when planning a root canal treatment for a mandibular first molar. Careful clinical and radiographic examination is indispensable in the diagnosis of any anatomic variation in the root canal system of any tooth prior to initiating treatment.

As this study revealed that the incidence of a third root in Palestinian population was within the range of previous reports from the Middle East but considerably lower than the percentage from the Far East, the use of conventional two-dimensional radiographs for the assessment of RE would be probably considered as a limitation in the clinical approach and methodology of this study. Hopefully, future research in Palestine would be able to study a larger and more varied population utilizing Cone Beam Computed Tomography.

## Figures and Tables

**Table 1:** Incidence of RE in subjects according to gender.

	Number of first molars examined	Number of RE	%
Males	137	5	3.64
Females	185	7	3.78

Total	322	12	3.73
